# Proteomic Profile Regulated by the Immunomodulatory Jusvinza Drug in Neutrophils Isolated from Rheumatoid Arthritis Patients

**DOI:** 10.3390/biomedicines12122740

**Published:** 2024-11-29

**Authors:** Mabel Hernández-Cedeño, Arielis Rodríguez-Ulloa, Yassel Ramos, Luis J. González, Anabel Serrano-Díaz, Katharina Zettl, Jacek R. Wiśniewski, Gillian Martinez-Donato, Gerardo Guillen-Nieto, Vladimir Besada, María del Carmen Domínguez-Horta

**Affiliations:** 1 Autoimmunity Project, Department of Pharmaceuticals, Biomedical Research Division, Center for Genetic Engineering & Biotechnology (CIGB), Havana 10600, Cuba; mabel.hernandez@cigb.edu.cu (M.H.-C.); anabel.serrano@cigb.edu.cu (A.S.-D.); 2 Mass Spectrometry Laboratory, Proteomics Group, Department of System Biology, Biomedical Research Division, Center for Genetic Engineering & Biotechnology (CIGB), Havana 10600, Cuba; yassel.ramos@cigb.edu.cu (Y.R.); luis.javier@cigb.edu.cu (L.J.G.); vladimir.besada@cigb.edu.cu (V.B.); 3 Biochemical Proteomics Group, Department of Proteomics and Signal Transduction, Max-Planck Institute of Biochemistry, 82152 Munich, Germany; zettl@biochem.mpg.de (K.Z.); jwisniew@biochem.mpg.de (J.R.W.); 4 Biomedical Research Division, Center for Genetic Engineering & Biotechnology (CIGB), Havana 10600, Cuba; gillian.martinez@cigb.edu.cu (G.M.-D.); gerardo.guillen@cigb.edu.cu (G.G.-N.); 5 Latin American School of Medicine, Havana 19108, Cuba

**Keywords:** proteomics, CIGB-814, CIGB-258, Jusvinza, inflammation, NETosis

## Abstract

Jusvinza is an immunomodulatory drug composed of an altered peptide ligand (APL) designed from a novel CD4+ T cell epitope of human heat shock protein 60 (HSP60), an autoantigen involved in the pathogenesis of rheumatoid arthritis (RA). The peptide induces regulatory T cells and decreases levels of TNF-α and IL-17; pre-clinical and phase I clinical studies support its use for the treatment of RA. This peptide was repositioned for the treatment of COVID-19 patients with signs of hyperinflammation. Neutrophils play a pathogenic role in both RA and severe forms of COVID-19. To add novel evidence about the mechanism of action of Jusvinza, the proteomic profile regulated by this peptide of neutrophils isolated from four RA patients was investigated using LC-MS/MS and bioinformatics analysis. A total of 149 proteins were found to be differentially modulated in neutrophils treated with Jusvinza. The proteomic profile regulated by Jusvinza is characterized by the presence of proteins related to RNA splicing, phagocytosis, endocytosis, and immune functions. In response to Jusvinza treatment, several proteins that regulate the NF-κB signaling pathway were differentially modulated, supporting the peptide’s anti-inflammatory effect. Proteins related to metabolic pathways that supply ATP for cellular functions or lipid metabolites with immunoregulatory properties were also identified. Additionally, several structural components of neutrophil extracellular traps (NETs) were decreased in Jusvinza-treated cells, supporting its impairment of this biological process. Of note, these findings were validated by in vitro experiments which confirmed that Jusvinza decreased NET formation. Such results provide evidence of the molecular mechanism of action and support the therapeutic potentialities of Jusvinza to treat other diseases characterized by hyperinflammation besides RA and COVID-19.

## 1. Introduction

Induction of immunological tolerance is an interesting therapeutic approach for autoimmune diseases such as rheumatoid arthritis (RA). In relation to this, we designed and developed a novel drug, called Jusvinza, for the control of inflammation associated with RA. The active ingredient of Jusvinza is an altered peptide ligand (APL). The APL closely resembles immunogenic peptides, but it contains one or two substitutions at the specific sites where they interact with T cell receptor or class II HLA molecules. These substitutions have a significant impact on the pathway involved in T cell activation. Among the autoantigens involved in the pathogenesis of RA, we specifically selected the 60 kDa cell stress protein (HSP60) for the design of the APL. This selection was based on the therapeutic potential of HSP60 in the treatment of autoimmune diseases. Our hypothesis was that an APL derived from HSP60 would enhance these therapeutic effects [1]. The HSP60-derived APL was designed using bioinformatics tools. First, a new epitope for T cells was identified in the N-terminal region of the sequence of human HSP60. Then, this epitope was modified in a single amino acid to increase its affinity to the class II HLA molecules associated with RA [2].

Pre-clinical and clinical studies in RA indicated that Jusvinza is safe and reduces inflammation, without causing immunosuppression [3,4]. Recently, the Cuban agency for drug control (CECMED) approved Jusvinza’s medical registration [5]. Jusvinza reduced pro-inflammatory cytokines in several experimental systems [6] as well as in two animal models of RA [2,4]. In addition, it reduced IL-6 levels similarly to tocilizumab in a systemic inflammation model in Zebrafish [7]. Also, Jusvinza treatment reduced antibodies against cyclic citrullinated peptides (anti-CCPs) in RA patients [8]. It is known that NETosis, which is triggered by contact with environmental microorganisms, is the leading source of citrullinated autoantigens in RA [9]. Anti-CCPs, together with rheumatoid factors, precede the onset of disease symptoms and can be used to predict a more severe disease course, indicating their pathogenic role in RA. Neutrophils also play a crucial role in the initiation and progression of RA [10].

In RA disease, both blood and synovial fluid neutrophils have an increased capacity to produce ROS [11,12]. This leads to a microenvironment at joint surfaces with elevated levels of ROS, proteases, and cytotoxic factors, causing damage to underlying tissues [13]. In addition, neutrophil granule proteases not only damage collagen fibers within cartilage but also activate proteins such as matrix metalloproteinases, cytokines, and chemokines [13,14,15]. In addition, ROS production within a joint disrupts the oxidative balance and influences adaptive immune responses in the synovial environment [16]. ROS-induced activation of NF-κB in synovial fibroblasts triggers the production of pro-inflammatory prostaglandins by cyclooxygenase-2 [17]. ROS also affect the local T cell population, contributing to differentiation towards TH17 and TH1 [18]. Neutrophil-derived cytokines and chemokines play a critical role in regulating both innate and adaptive immune responses in RA [15]. Neutrophil apoptosis is also dysregulated in RA, leading to delayed apoptosis within the synovial joints. This contributes to chronic inflammation, immune cell recruitment, and prolonged release of proteolytic enzymes [19,20].

On these bases, Jusvinza was repositioned in our country for the treatment of hyperinflammation that distinguishes patients with COVID-19 and received an Emergency Use Authorization from CECMED [21]. Congruently, in a clinical setting, Jusvinza reduced the inflammation that characterizes COVID-19 patients by decreasing levels of IL-6, TNFα, and IL-10; restoring the physiological serum levels of autoreactive T-lymphocytes; and inducing Treg cells [22]. Activated Treg cells could migrate to inflammation sites and cross-recognize the original epitope of HSP60, which increases its concentration in endothelial tissue, thus inhibiting autoimmune damage to endothelia caused during viral infection [23]. The clinical improvement of COVID-19 patients treated with Jusvinza is also associated with a decrease in several coagulation and inflammation biomarkers [24], including calprotectin. The latter, being the most-abundant cytosolic protein derived from neutrophils, is found at high blood concentrations during inflammatory processes [25,26,27].

Neutrophils are specialized cells of the innate immune system that play a key physiological role in host defense against microorganisms. Currently, three mechanisms have been described by which neutrophils remove pathogens and prevent their dissemination throughout the body. These include phagocytosis, degranulation, and NETosis. Phagocytosis is initiated by the internalization of targeted organisms or particles and enables the effective elimination of pathogens. Neutrophil degranulation involves the sequential release of different granule types containing antimicrobial and cytotoxic proteins [28]. Also, neutrophils can secrete several pro-inflammatory cytokines and molecules associated with inflammation [29]. NETosis is the formation of neutrophil extracellular traps (NETs). NETs, composed of DNA, histones, and proteins from granules and cytoplasm, immobilize and kill microorganisms, thereby maintaining host defense [28]. Nevertheless, dysregulated neutrophil activation contributes to the pathogenesis of inflammatory diseases, such as RA [30] and COVID-19 [31].

For all these reasons, we determined the total cell proteome regulated by Jusvinza using a primary culture of non-pooled neutrophils which were isolated from four RA patients. Using label-free quantitative proteomics, the molecular perturbations induced by this immunomodulatory peptide were described in neutrophils after 6 h and 18 h of treatment. Importantly, NETosis was validated as a biological process that is counteracted in the presence of Jusvinza. In general, these results provide molecular support to explain the anti-inflammatory effect of Jusvinza and indicate its therapeutic potentialities.

## 2. Materials and Methods

### 2.1. Patients and Neutrophil Isolation

Cells from four RA patients with similar demographic characteristics were used in this study. All patients had moderate disease activity according to their DAS 28 disease scores (4.93 ± 0.09). They were all white women and aged between 50 and 59 (Table 1). Neutrophil isolation was carried out under sterile conditions using a procedure reported by Nauseef et al. [32]. In brief, 20 mL EDTA blood samples were collected from the RA patients and added to 3% of dextran solution (18–20 min at room temperature). Leukocyte-rich plasma was collected and centrifuged for 10 min at 500× *g* and 4 °C. The cellular pellet was resuspended in 10 mL of phosphate-buffered solution (PBS) and transferred onto Ficoll–Paque separation medium. Differential centrifugation was performed at 200× *g* for 30 min and 4 °C. The collected granulocytes were treated with 0.2% and 1.6% NaCl solutions to remove residual erythrocytes. Finally, neutrophils were dissolved in RPMI 1640 medium, and cell counts were performed using a Neubauer chamber.

### 2.2. Sample Preparation

For proteomic analysis, neutrophils isolated from the four RA patients (10^6^ cells per condition, four biological replicates) were independently incubated with 11.5 µM of the peptide Jusvinza for 6 h or 18 h. In parallel with the Jusvinza-treated groups, non-treated neutrophils were incubated for 6 h or 18 h with a vehicle and used as normalization controls. Total proteins from each replicate of the Jusvinza-treated and non-treated neutrophils were extracted with 1.5% SDS and 50 mM DTT and boiling conditions for 10 min. Total protein extracts were then processed by multienzyme digestion filter-aided sample preparation (MED-FASP) with overnight Lys-C and tryptic digestions [33]. Protein and peptide concentrations were estimated by a tryptophan fluorescence-based assay previously described in [34]. Finally, 1 µg of peptides for each sample was injected for nanoLC-MS/MS analysis.

### 2.3. NanoLC-MS/MS

A NanoLC EASY-nLC 1200 system coupled to a Q-Exactive HF mass spectrometer (Thermo Scientific, Waltham, MA, USA) was used. Chromatographic runs for Lys-C and trypsin-derived peptides were performed in a home-made column (Dr. Maisch ReproSil-Pur C18-AQ 1.9 μm, 75 μm ID, 20 cm length) thermostated at 60 °C. Peptides were eluted at 300 nL/min with a 120 min solution B (A: 0.1% formic acid in water; B: 0.1% formic acid in acetonitrile) gradient, starting at 5% solution B up to 30% for 95 min, then increased to 60% B for 5 min, and finally up to 95% B for a further 5 min. A voltage of 2 kV was applied to the column tip to induce the nanospray, and the mass range 300–1650 *m*/*z* was scanned in data-dependent acquisition mode. Each mass spectrum obtained at a resolution of 60,000 (*m*/*z* 200, 20 ms injection time) was followed by 15 MS/MS spectrum runs (28 ms injection time) at a resolution of 15,000 (*m*/*z* 200). Proteins were only considered when detected in at least three replicates in any of the groups.

### 2.4. Data Processing

Identification of peptides and proteins was based on the match-between-runs procedure using MaxQuant software (version 1.6.2.10) [35], considering oxidation (M), deamidation (NQ), and N-terminal acetylation as variable modifications. Alignment of chromatographic runs was allowed with default parameters (20 min time window and a matching of 0.7 min between runs). Filtering and quantification were performed using the Perseus computational platform (version 1.6.2.2) [36]. The Student’s *t*-test was employed to identify statistically significant changes (*p*-values lower than 0.05) in protein levels, after filtering for two valid values in at least one group. An additional cutoff of a 1.5-fold change between the Jusvinza-treated neutrophils and the non-treated controls was also applied.

### 2.5. Bioinformatics Analysis

Proteins with similar patterns of expression levels were clustered using the k-means algorithm implemented in MultiExperiment Viewer (MeV) (version 4.5.1) [37]. The figure of merit (FOM) that estimates the predictive power of a clustering algorithm [38] was calculated to suggest the appropriate input parameters for k-means. Protein clusters were visualized using the Matrix2png software tool (version 1.2.3) (https://matrix2png.msl.ubc.ca/index.html, accessed on 20 June 2024) [39]. 

Biological processes, cellular components, and biological pathways significantly represented in differentially modulated proteins were identified through functional annotation and enrichment analysis, based on the information annotated in the Gene Ontology (GO; http://www.geneontology.org, accessed on 20 June 2024), KEGG PATHWAY (https://www.genome.jp/kegg/, accessed on 20 June 2024), and Reactome (https://reactome.org/, accessed on 20 June 2024) databases [40,41,42,43]. Analysis was performed with the Metascape gene annotation and analysis resource (https://metascape.org/, accessed on 6 November 2020), a web-based tool that computes accumulative hypergeometric distributions and enrichment factors to identify significantly enriched biological processes through statistical analysis (*p*-value < 0.01, enrichment factor > 1.5) [44]. For the proteomic profiles regulated at 6 h or 18 h after Jusvinza treatment, the Metascape Custom Analysis option was selected [accessed on 5 November 2020] and the Homo sapiens genome was used as background. Down- and up-regulated proteins at 6 h were used as independent input datasets for meta-analysis, while modulated proteins at 18 h were analyzed as a single file.

In addition, interactions among differentially modulated proteins were retrieved using the STRING database (http://string-db.org/; accessed on 13 June 2023) [45]. Besides the proteomic profile regulated in neutrophils isolated from the four RA patients included in the study, those proteins that were differentially modulated in neutrophils from at least three RA patients were also included for protein interaction network analysis. In these analyses, all STRING interaction sources were selected, the confidence score was fixed at 0.7, and a 2nd shell of interactors, with no more than 50 additional proteins, was allowed for network representation. Biological processes and protein complexes represented in protein–protein interaction networks were retrieved through a search of the literature and the STRING functional enrichment tool. All biological networks (functional and protein–protein interaction networks) were visualized using Cytoscape software (version 3.9) [46].

### 2.6. NETosis Detection

Neutrophils (2 × 10^5^ cells/mL) isolated from three healthy donors were cultured for 30 min at 37 °C in 24-well plates. Then, LPS from Escherichia coli (150 μg/mL, strain: O111:B4) was added to the culture. Neutrophils alone or with Jusvinza or LPS were used as controls. In addition, 5 μg/mL of Jusvinza was added with LPS or 30 min later. After, the cultures were incubated for 3 h at 37 °C and 5% CO_2_.

NET quantification was performed according to a modified method to calculate the nuclear area expansion [47]. In brief, after NET induction in 24-well plates, neutrophils were fixed in 4% paraformaldehyde, treated with 0.5% Triton X-100, and washed two times with PBS. Then, DNA was stained with 10 μg/mL propidium iodide solution, and the plates were incubated in the dark for 30 min at room temperature. The section fluorescence was quantified using ImageJ software (version 1.48). Five fields per well (four extremes and the center) were captured at 20× magnification for each experimental condition. Neutrophils in NETosis were defined when the nucleus area was ≥1-fold the cellular area. The quantity of NETs was expressed as a proportion (the number of neutrophils in NETosis divided by the total number of neutrophils) in five fields for each experimental condition.

For the in vitro NET study, the data are expressed as the means ± SDs (the number of neutrophils in NETosis: the total number of neutrophils) from three independent experiments with duplicate samples. Significant differences were calculated by one-way ANOVA followed by Tukey’s multiple comparisons test.

## 3. Results

### 3.1. Profiling the Jusvinza-Regulated Proteome in Neutrophils of RA Patients

**Figure 1:**
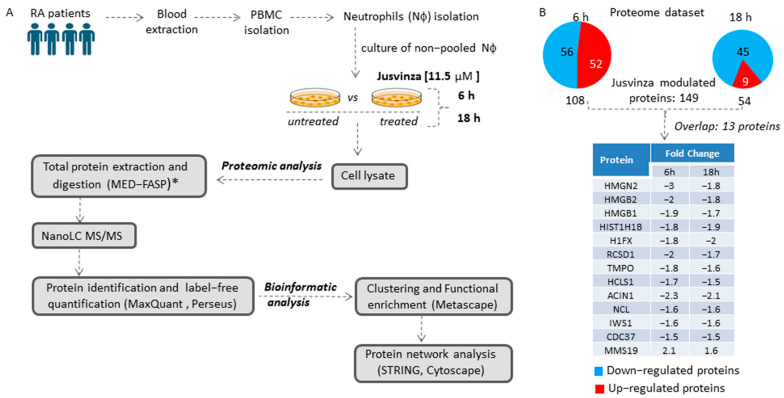
Proteomic profile of neutrophils treated with Jusvinza. (**A**) Workflow for the exploration and analysis of the proteomic profile modulated in response to Jusvinza treatment. (**B**) Number of significantly modulated proteins in Jusvinza-treated neutrophils at 6 h and 18 h. Down- and up-regulated proteins are highlighted in blue and red, respectively. The table shows the overlapping proteins between the two datasets. (*) MED-FASP: multienzyme digestion filter-assisted sample preparation [33].

To identify the array of proteins regulated by Jusvinza, a label-free quantitative proteomic analysis of neutrophils isolated from four RA patients was performed (Figure 1A). Isolated neutrophils (from each patient’s biological sample) were treated or not with 11.5 µM of Jusvinza. A total of 4092 proteins were identified in the Jusvinza-treated neutrophils (Supplementary Table S1). After 6 h and 18 h of Jusvinza treatment, 108 and 54 proteins were significantly modulated in neutrophils derived from the four patients, respectively (Figure 1B, Supplementary Table S1). In neutrophils treated with Jusvinza for 6 h, the number of down- and up-regulated proteins was quite similar (Figure 1B). In contrast, most of the differentially modulated proteins at 18 h were down-regulated after peptide treatment.

The differentially modulated proteins were clustered according to their fold changes using the k-means algorithm. As a result, six protein clusters were identified, three of which contained the majority of differentially modulated proteins at 6 h or 18 h after Jusvinza treatment (Supplementary Figure S1). Proteins that overlapped between the two datasets were clustered together; most of them were down-regulated, with very similar expression levels at both time points (Supplementary Figure S1, Figure 1B).

### 3.2. Functional Characterization of the Jusvinza-Regulated Proteome

**Figure 2:**
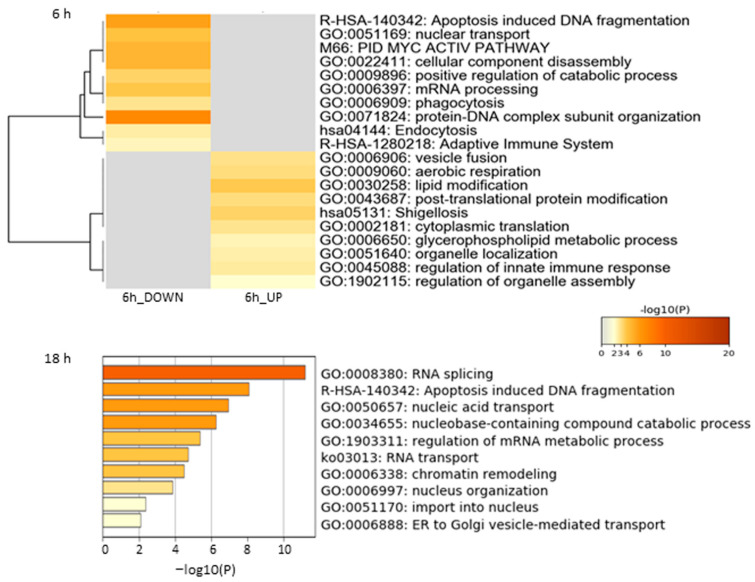
Enrichment analysis of differentially modulated proteins in neutrophils treated with Jusvinza at 6 h and 18 h. Biological processes and pathways significantly represented in the proteomic profiles (*p*-value < 0.01, enrichment factor > 1.5) were identified using the Metascape gene annotation and analysis resource (https://metascape.org/, accessed on 5 November 2020). In the heatmap and bar graph, enriched terms are colored according to *p*-values.

**Figure 3:**
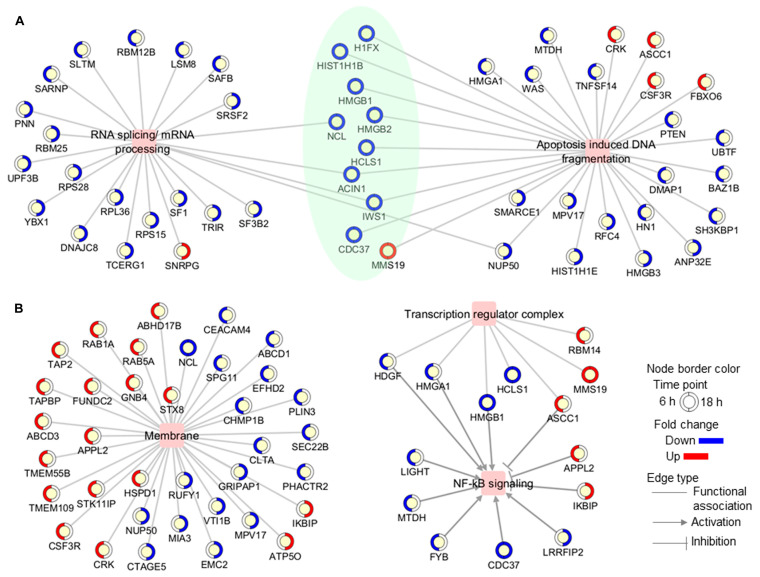
Functional association networks between differentially modulated proteins and some (**A**) biological processes and (**B**) subcellular locations which were found to be over-represented in the proteomic profile. In networks, proteins are shown as yellow circles, with the outside circle representing the expression level (blue, decreased; red, increased; white, not differentially modulated) and colored in a clockwise fashion according to the fold change at each time point (6 h and 18 h). Proteins that were modulated at both time points are highlighted in green.

Functional enrichment analysis of the Jusvinza-regulated proteome in neutrophils was performed using the Metascape bioinformatics tool [44]. In this analysis, protein–DNA complex subunit organization, phagocytosis, endocytosis, and the adaptive immune system were identified as biological processes or pathways significantly represented in the proteomic profile down-regulated in neutrophils treated with Jusvinza for 6 h (Figure 2, Supplementary Table S2). As shown in Figure 2, a different array of biological processes was found to be over-represented in the Jusvinza up-regulated proteome, including aerobic respiration, lipid modification, cytoplasmic translation, and regulation of the innate immune response. The proteomic profile regulated at 18 h in Jusvinza-treated cells was distinctively characterized by the over-representation of functional categories related to intracellular transport, such as nucleic acid transport and ER-to-Golgi vesicle-mediated transport (Figure 2, Supplementary Table S2). Of note, proteins involved in RNA splicing and apoptosis-induced DNA fragmentation were found to be differentially regulated in Jusvinza-treated neutrophils irrespective of the treatment time. These biological processes include most of the differentially modulated proteins that were regulated at 6 h and 18 h in response to Jusvinza treatment (Figure 3A).

On the other hand, proteins located in membrane structures, including lysosomal and endosomal membranes, were significantly represented in the Jusvinza-regulated proteome (Supplementary Figure S2, Supplementary Table S2). In fact, 26 (24%) and 11 (20%) membrane proteins were differentially modulated by Jusvinza at 6 h and 18 h, respectively (Figure 3B). Additionally, the transcription regulator complex was found to be over-represented in the proteomic profile regulated at 6 h by Jusvinza (Supplementary Figure S2, Supplementary Table S2). In line with this result, transcriptional factors (SAFB, IWS, FLI1, and YBX1) were also differentially modulated in Jusvinza-treated neutrophils. Importantly, four proteins of the transcription regulator complex (HDGF, HMGA1, HMGB1, and ASCC1) are associated with the NF-κB signaling pathway. As illustrated in Figure 3B, the Jusvinza-regulated proteome provides additional evidence suggesting that NF-κB regulation could mediate the neutrophil response to the peptide treatment. Positive regulators of NF-κB signaling (LIGHT, MTDH, FYB, CDC37, and LRRFIP2) were down-regulated in Jusvinza-treated cells, while negative regulators (APPL2 and IKBIP) were identified with increased abundance levels irrespective of the treatment time. Of note, two of these proteins were among the most up- and down-regulated by Jusvinza (LIGHT: FC = −5.6, IKBIP: FC = 3.2; Supplementary Table S1).

**Figure 4:**
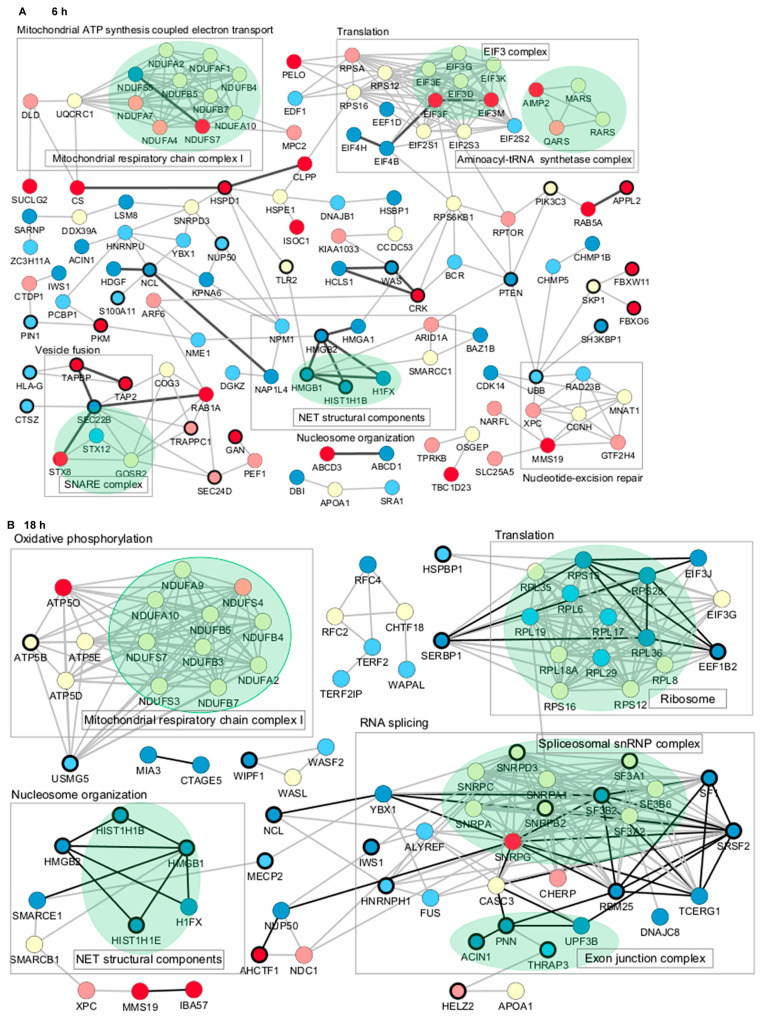
Protein–protein interaction networks associated with the proteomic profile modulated at 6 h (**A**) and 18 h (**B**) in Jusvinza-treated neutrophils. In both networks, proteins are represented according to the expression level (blue, decreased; red, increased; yellow, not identified); dark and light colors represent proteins identified in neutrophils isolated from four and three AR patients, respectively. Biological processes and proteins complexes gathered using the STRING functional enrichment tool and datamining are indicated by squares and green colors, respectively. Proteins related to the immune system are highlighted by a bold circle. Direct interactions between differentially modulated proteins are represented by black edges.

To further characterize the proteome profile regulated by Jusvinza, the interaction network of differentially modulated proteins was represented using the STRING database [45]. A total of 23 and 45 direct interactions connected only 29 and 30 proteins differentially modulated at 6 h and 18 h, respectively (Figure 4). Therefore, the network was expanded to include additional proteins connecting the regulated ones. Most of the connecting proteins were identified in neutrophils isolated from at least three of the four patients who were included in the proteomic study. Differentially modulated proteins with the largest number of interactions included the following: the 60 kDa heat shock protein (HSPD1), the phosphatase and tensin homolog (PTEN), high mobility group protein B1 (HMGB1), and plasminogen activator inhibitor 1 RNA-binding protein (SERBP1) (Figure 4). Such proteins, which have a high degree of network connection, might exert regulatory functions in response to Jusvinza treatment. As illustrated in Figure 4, proteins related to the immune system (black-bordered nodes) were dispersed over the protein interaction network perturbed at 6 h and 18 h after treatment with Jusvinza, demonstrating the relationship between the proteomic dataset and the biological system under study. On the other hand, clusters of proteins related to mitochondrial respiratory chain complex I, the EIF3 complex, the SNARE complex, the spliceosomal snRNP complex, and the exon junction complex include Jusvinza-modulated proteins (Figure 4). In line with these results, the biological processes of aerobic respiration, cytoplasmic translation, endocytosis, and RNA splicing were identified through functional enrichment analysis (Figure 2, Supplementary Table S2). Importantly, the array of proteins related to nucleosome organization appeared to be modulated at 6 h and 18 h after treatment, suggesting that these proteins play an essential role in the molecular events mediated by the peptide.

### 3.3. Effect of Jusvinza on NETosis

**Figure 5:**
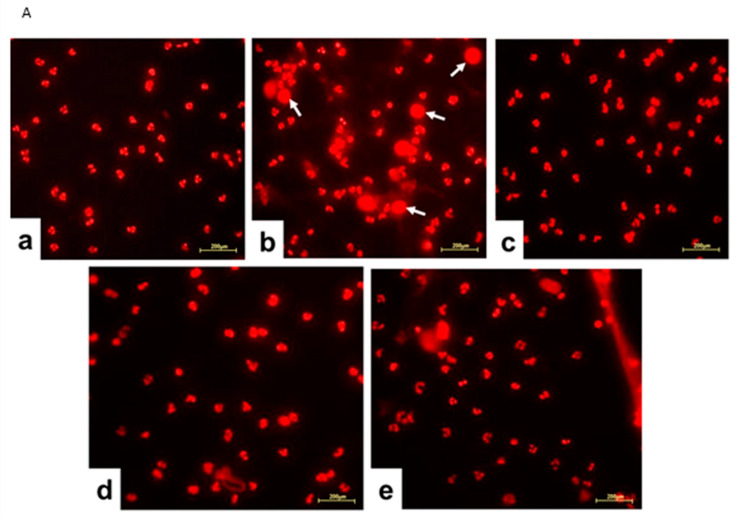

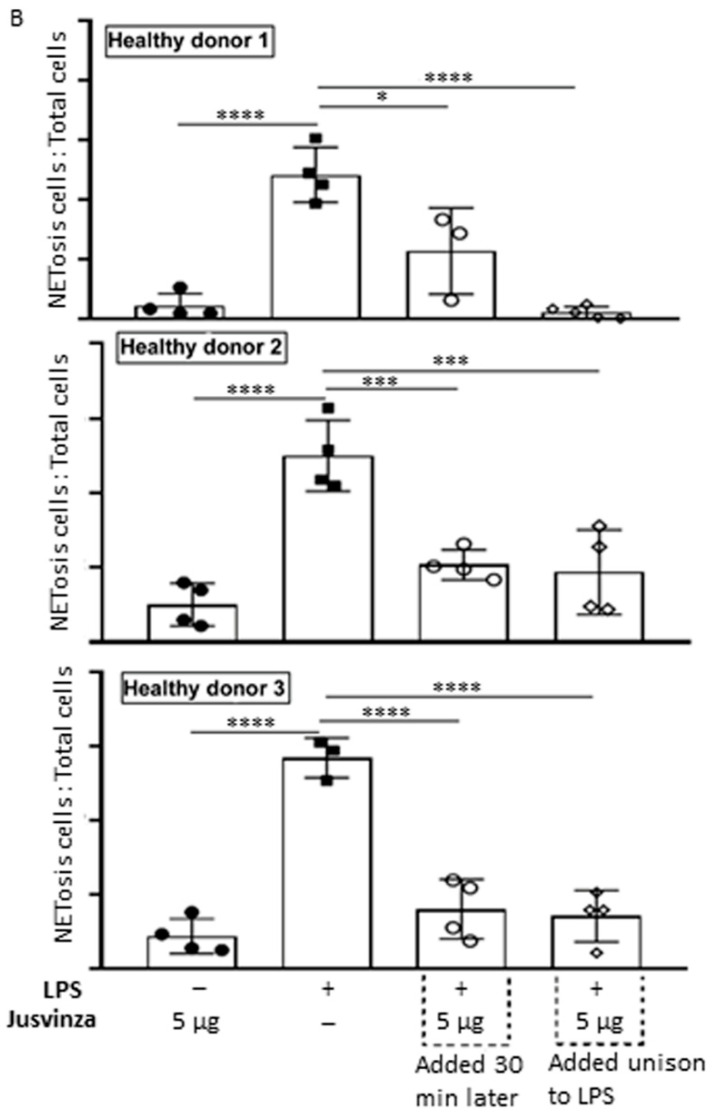
(**A**) Representative fluorescence images of LPS-induced NETosis. Neutrophils isolated from healthy donors were subjected to different experimental conditions: (**a**) unstimulated neutrophils, (**b**) LPS-stimulated neutrophils, (**c**) neutrophils stimulated with 5 μg of Jusvinza, (**d**) neutrophils simultaneously stimulated with LPS and 5 μg of Jusvinza, (**e**) neutrophils treated with 5 μg of Jusvinza 30 min after pre-stimulation with LPS. The DNA was stained with 10 μg/mL propidium iodide solution. Images were captured at 20× magnification. White arrows indicate neutrophils in NETosis. (**B**) Quantification of NETs induced by LPS in neutrophils treated with Jusvinza. NETs were quantified using neutrophils isolated from three healthy donors. Five fluorescent images were acquired for each experimental condition. Neutrophils in NETosis were defined when the nucleus area was ≥1-fold the cellular area. Results are expressed as a fraction of the number of neutrophils in NETosis/the total number of neutrophils. Significant differences were calculated by one-way ANOVA followed by Tukey’s multiple comparisons test (* *p*-value < 0.05, *** *p*-value < 0.0002, **** *p*-value < 0.0001).

Considering that the proteomic analysis evidenced that Jusvinza treatment decreases the abundance level of different NET structural components after 6 h and 18 h (Figure 4), we sought to determine whether Jusvinza inhibits NETosis. Morphological transformations in neutrophils during NETosis induction were identified by fluorescence microscopy (Figure 5A). LPS-stimulated neutrophils showed an increase in the area stained by propidium iodide (Figure 5A(b)). On the contrary, unstimulated (negative control) and Jusvinza-stimulated neutrophils retained their characteristic morphology with their lobed nuclei (Figure 5A(a,c)). Furthermore, the effect of Jusvinza on the morphological changes in neutrophils stimulated with LPS was studied when it was added to the culture or 30 min after having added the LPS (Figure 5A(d,e)).

In vitro, LPS-induced NETs were quantified using neutrophils isolated from three healthy donors. Figure 5B shows the results as the proportion of neutrophils in NETosis relative to the total number of cells. LPS significantly induced NETs (*p* < 0.0001) compared to neutrophils cultured without LPS. Jusvinza significantly reduced NETs when co-added with LPS. Likewise, treatment with Jusvinza 30 min after LPS stimulation of neutrophils significantly reduced NETs.

## 4. Discussion

Jusvinza is based on a classic APL derived from HSP60, which has demonstrated therapeutic effects in RA patients and also in COVID-19 patients with signs of hyperinflammation [8,23,48]. The mechanism of action of Jusvinza is not fully understood. Here, we used label-free quantitative proteomics to identify the Jusvinza-regulated proteome and explore the molecular perturbations promoted by this anti-inflammatory peptide in neutrophils following 6 h and 18 h of treatment. A total of 149 proteins were differentially modulated by Jusvinza treatment, including membrane proteins and low-abundance transcription factors, which are protein classes that usually have low coverage in proteomics datasets. These results demonstrate the usefulness of MED-FASP as a sample preparation method for proteomics analysis [49]. An array of 13 proteins, which are mainly related to RNA splicing or apoptosis-induced DNA fragmentation, were regulated by the peptide at both incubation times (6 h and 18 h). Additionally, the proteomic profile regulated at 6 h in Jusvinza-treated neutrophils was characterized by the presence of proteins related to phagocytosis, endocytosis, lipid metabolism, and immune functions. In comparison with the proteome modulated at 6 h, the effect of Jusvinza seemed to be dispersed at 18 h following treatment (only 54 proteins were differentially modulated). Probably, signal propagation to extracellular events could orchestrate the response to Jusvinza over longer treatment times (18 h and longer times were not covered in this quantitative proteomic experiment).

During the immune system response, neutrophils translocate to sites of infection or acute injury and sequester particulate substrates of microbial or endogenous origin via phagocytosis [50]. Importantly, the hematopoietic lineage cell-specific protein (HCLS1), which mediates neutrophil-directed migration [51,52], was down-regulated at 6 h and 18 h after Jusvinza treatment. In addition, the expression of phosphatase and tensin homolog protein (PTEN), which “prioritizes” neutrophil chemotaxis toward end-target chemoattractants and prevents “distraction” by chemokines during migration [53], decreased at 6 h in response to Jusvinza treatment. PTEN antagonizes the PI3K-AKT signaling pathway; such regulation triggers different effects in the immune system response depending on the cell type [54,55]. Chemotaxis of neutrophils is a cellular process that also depends on cytoskeleton remodeling. Supporting the hypothesis that Jusvinza should hamper neutrophil migration to sites of inflammation, besides down-regulation of HCLS1 and PTEN, the expression of two regulatory proteins of the actin cytoskeleton decreased in peptide-treated cells: Wiskott–Aldrich syndrome protein (WASP) and WAS-interacting protein family member 1 (WIP). On the other hand, proteins related to phagocytosis, which is an essential effector function of the neutrophil-mediated immune response, were down-regulated at 6 h in response to Jusvinza treatment. For instance, the expression of carcinoembryonic antigen-related cell adhesion molecule 4 (CEACAM) decreased eight-fold in response to Jusvinza treatment. CEACAM is a granulocyte orphan receptor that triggers the efficient phagocytosis of attached particles [56]. Based on this result, the influence of Jusvinza on the phagocytic ability of neutrophils should be validated in further experiments. Likewise, proteins related to endocytosis, like clathrin light chain A (CLTA), were down-regulated at 6 h in neutrophils treated with Jusvinza. CLTA is a main structural component of coated pits and vesicles during receptor-mediated endocytosis. Clathrin-mediated endocytosis regulates signal transduction pathways, leading to neutrophil polarization [57], priming of polymorphonuclear neutrophils (PMNs) [58], and priming of respiratory burst activity through ROS-mediated control of granule exocytosis [59]. These events modulate and enhance the neutrophil response at the site of inflammation. Altogether, the down-regulation of proteins related to migration, phagocytosis, and priming of neutrophils constitutes experimental evidence supporting the proposition that Jusvinza could decrease the over-activation of neutrophils, a condition linked to several pathologies, including RA. It would be interesting for future experiments to carry out a proteomic study to evaluate the effect of Jusvinza on monocytes and macrophages. These cells derive from the same myeloid lineage as neutrophils (myelomonocytic stem cells) and also contribute to the pathogenesis of RA [60,61].

Proteins that regulate metabolic functions were identified in this study. Lipid modification and glycerophospholipid metabolism were enriched in the proteomic profile up-regulated at 6 h in response to Jusvinza treatment. Of note, D-beta-hydroxybutyrate dehydrogenase mitochondrial (BDH1) and lysophospholipid acyl transferase 7 (MBOAT7), which are enzymes involved in the synthesis of anti-inflammatory metabolites, were up-regulated in Jusvinza-treated neutrophils. BHD1 catalyzes the interconversion of the two major ketone bodies produced during fatty acid catabolism: acetoacetate and β-hydroxybutyrate (BHB); the latter inhibits NLRP3 inflammasome activation in neutrophils and macrophages and subsequent IL-1 production [62,63]. MBOAT7 negatively regulates free arachidonic acid levels and synthesis of pro-inflammatory leukotriene B4 (LTB4) in neutrophils by catalyzing the conversion of lysophosphatidylinositol into phosphatidylinositol in the phospholipid remodeling pathway (the reacylation step) [64,65]. In addition, perilipin-3 (PLIN3), a protein related to lipid droplet (adiposome) biogenesis and prostaglandin E2 (PGE2) production [66], was down-regulated in response to Jusvinza treatment. PGE2, similar to LTB4, is a lipid mediator produced from arachidonic acid and possesses context-dependent immunoregulatory properties [67]. The molecular functions mediated by BDH1, MBOAT7, and PLIN3 illustrate the close interconnection between the metabolic and immune systems. Therefore, modulation of such proteins in response to Jusvinza treatment suggests that the peptide could drive cellular metabolic reprogramming to exert its immunoregulatory effects. Supporting such a hypothesis, proteins related to fatty acid beta oxidation (ABCD1, ABCD3, ILVBL, and DBI) and aerobic respiration (glycolysis: PKM; the TCA cycle: CS and SUCLG2; mitochondrial respiratory chain complex I: NDUFS7 and NDUFS5) were also modulated in Jusvinza-treated neutrophils. Recent results demonstrate that Jusvinza interacts with apolipoprotein A-I (APOA1) [7,68], the major protein component of high-density lipoprotein (HDL) in plasma. Notably, as demonstrated by network analysis, APOA1 is functionally related to acyl CoA-binding protein (DBI/ACBP), which was down-regulated by Jusvinza. DBI functions as an intracellular carrier of acyl-CoA esters [69] and promotes the expression of genes encoding key enzymes related to glycerolipid, cholesterol, and fatty acid metabolism [70]. DBI as well as APOA1 are annotated in the PPAR signaling pathway according to the KEGG database (KEGG_ID: hsa03320). Besides regulating the cellular response to lipids, the activation of PPARs (peroxisome proliferator-activated receptors) has anti-inflammatory effects on immune cells [71]. Specifically, PPARγ promotes neutrophil apoptosis and clearance during the resolution phase of the inflammatory response [72]. The proteomic profile modulated at 18 h following treatment with Jusvinza includes the protein helicase with zinc finger domain 2 (HELZ2), which functions as a transcriptional activator of nuclear receptors, including PPARα and PPARγ [73]. Importantly, HELZ2, as part of the PPARA coactivator complex, mediates the transcription of APOA1, according to the Reactome database (Reactome ID: R-HSA-1989754). The expression level of HELZ2 increased in Jusvinza-treated neutrophils isolated from three of the four AR patients who were included in the study. The functional association of APOA1, DBI, and HELZ2 with the PPAR signaling pathway, as well as the modulation of proteins related to metabolic pathways that supplies ATP for cellular functions or lipid metabolites with immunoregulatory properties, supports the idea that regulation of metabolism could play an important role in the molecular mechanism of Jusvinza.

As we mentioned before, proteins related to transcription were also modulated in the presence of Jusvinza. Among the transcription factors, the protein IWS1 homolog (IWS1) was down-regulated at 6 h and 18 h, while nuclease-sensitive element-binding protein 1 (YBX1) and the transcriptional regulator ERG (FLI1) were both down-regulated at 18 h after Jusvinza treatment. IWS1, a transcription factor essential for cell proliferation, defines the composition of the RNA polymerase II complex and modulates the production of mature mRNA transcription [74,75]. FLI1 regulates the expression of metalloproteases (MMP-1, MMP-3, and MMP-10) and the pro-inflammatory cytokine IL-10; therefore, during chronic inflammatory conditions, such as AR and atherosclerosis, FLI1 down-regulation could attenuate tissue damage associated with inflammatory responses [76]. The transcription factor YBX1 has also been related to the inflammatory response. In addition to the effect on NF-kB transcriptional activity, in the early phase of inflammation, YBX1 positively regulates CCL5/RANTES chemokine expression [77,78]. YBX1 is secreted by immune cells and was found to be up-regulated during systemic inflammation and vascular damage [79,80]. The decreased expression levels of FLI1 and YBX1 in response to Jusvinza treatment are in agreement with the accumulated evidence demonstrating that the peptide induces an anti-inflammatory response [6,23,24].

In line with these findings, several proteins which regulate NF-κB signaling were modulated in Jusvinza-treated neutrophils. The NF-κB transcription factor is a master regulator of several biological processes, including the inflammatory response. Ligand binding to transmembrane receptors like TNFR, IL1R, and TLR promotes phosphorylation and concurrent activation of the I-kappa-B kinase complex (IKK). This complex promotes ubiquitination and proteosomal degradation of inhibitory IκB proteins, releasing the active NF-κB complex, which is translocated into the nucleus to regulate gene transcription [81]. In response to Jusvinza treatment, IKK interacting protein (IKIP) was up-regulated at 18 h. IKIP inhibits the phosphorylation of IKK and negatively regulates NF-κB activation and pro-inflammatory cytokine production [82]. Similarly, at 6 h following Jusvinza treatment, two proteins that negatively regulate NF-κB signaling were up-regulated: DCC-interacting protein 13-beta (APPL2) and activating signal cointegrator 1 complex subunit 1 (ASCC1). APPL2 is a multifunctional adaptor protein that regulates the LPS/TLR4 signaling pathway by inhibiting nuclear translocation of NF-κB p65 and suppressing inflammatory cytokine secretion [83]. On the other hand, ASCC1 inhibits the expression of NF-κB target genes [84]. A truncated and inactive variant of ASCC1 (p.S78*), which did not reduce the transcriptional activation of NF-κB, has been related to disease severity in RA patients [84]. Furthermore, proteins that activate NF-κB signaling decreased at 6 h and 18 h after Jusvinza treatment. Among these proteins, metadherin (MTDH) and the tumor necrosis factor ligand superfamily member 14 (TNFSF14/LIGHT) were down-regulated at 6 h in response to Jusvinza. MTDH activates the NF-κB pathway by facilitating the degradation of IκB and acting as a transcriptional co-activator of NF-κB p65 [85,86]. LIGHT is considered a pro-inflammatory cytokine that triggers activation of non-canonical NF-κB and STAT3 signaling after binding to receptors (TNFRSF3 or TNFRSF14) [87,88]. Increased levels of LIGHT have been detected in autoimmune and chronic inflammation diseases like Crohn’s disease, coronary disease, RA, and COVID-19 [89,90,91,92]. Specifically, LIGHT promotes RA-FLS (fibroblast-like synoviocyte) proliferation and induces a pro-inflammatory response characterized by increased levels of MCP-1, IL-8, MIP-1α, and ICAM-1 [89]. LIGHT also induces osteoclast differentiation and plays a critical role in the inflammatory joint destruction of RA patients [93]. In the case of COVID-19, LIGHT is related to cytokine release syndrome (CRS), and its expression levels are highly up-regulated in the sera of critical patients who require mechanical ventilation [91].

Other pieces of evidence supporting the impact of Jusvinza on NF-κB signaling were the down-regulation of the HSP90 co-chaperone Cdc37 (CDC37) and leucine-rich repeat flightless-interacting protein 2 (LRRFIP2). Both proteins play a positive role in regulating TLR-mediated NF-κB activation and cytokine production [94,95]. Together with the HSP90 chaperone, CDC37 is a functional component of the IKK complex, mediating its assembly and activation in response to TNF-mediated signaling [96]. The expression level of CDC37 decreased at 6 h and 18 h after Jusvinza treatment, while LRRFIP2 was down-regulated only at later times of incubation (18 h) with the peptide. Besides its role in NF-κB signaling, LRRFIP2 is required for NRL3 inflammasome activation in response to Ca^2+^ signaling [97]. Therefore, the results suggest that Jusvinza could orchestrate different mechanisms to attenuate the inflammatory process. Relatedly, heat shock factor binding protein 1 (HSBP1), which inhibits the transcriptional activity of heat shock factor 1 (HSF1) [98], was down-regulated in neutrophils in response to Jusvinza. The transcription factor HSF1 is known to inhibit the expression of pro-inflammatory cytokines and mediators (IL-1β, TNF-α, IL-6, HMGB1, and NF-κB) [99,100,101]. In addition, HSF1 functions as a transcriptional activator of the chaperone HSP60/HSPD1, the anti-inflammatory cytokine IL-10, and the zinc finger protein SNAI1, which reduces NLRP3 inflammasome activation [102,103,104]. Interestingly, HMGB1 and HSP60/HSPD1 proteins were down- and up-regulated in Jusvinza-treated neutrophils, respectively. A possible explanation of the differential regulation of HMGB1 and HSP60/HSPD1 might be the decreased levels of HSBP1 and the subsequent activation of the heat shock response (HSR) triggered by HSF1. Of note, HSR-inducing therapies may improve inflammatory profiles in COVID-19 patients with metabolic diseases (obesity and diabetes), which are comorbidities that increase the severity of SARS-CoV-2 infections [105].

Among proteins related to mRNA processing, several components of the spliceosome (SF3B2, SF1, RBM25, and SRSF2) and the exon junction (ACIN1, WIBG, PNN, and UPF3B) complexes were modulated by Jusvinza, including small nuclear ribonucleoprotein G (SNRPG; FC = 8.1), which was the most up-regulated protein at 18 h. Regulation of the splicing machinery could modulate innate immunity. For instance, the inhibition of serine/arginine-rich splicing factor 2 (SRSF2) reduces inflammatory cytokine levels [106], and this protein was down-regulated at 18 h by Jusvinza treatment. Furthermore, nucleolin (NCL), a protein that regulates the ribosome biogenesis pathway by several mechanisms, including the processing of pre-rRNA and ribosome assembly [107], was down-regulated at 6 h and 18 h in Jusvinza-treated neutrophils. In line with modulation of NCL, ribosomal proteins as well as translation initiation and elongation factors were down-regulated in the presence of Jusvinza. Although mainly localized in nucleoli, NCL is also found in cell membranes, promoting the inflammatory response through the internalization of LPS or immunogenic extracellular mitochondria DNA (mtDNA) [108,109]. Increased levels of mtDNA have been detected in synovial fluid samples from RA patients—an event correlated with join inflammation [110]. On the other hand, the expression of plasminogen activator inhibitor 1 RNA-binding protein (SERBP1) was decreased at 18 h in response to Jusvinza treatment. SERBP1 binds to a cyclic nucleotide-responsive sequence located in the 3′-untranslated region of PAI-1 mRNA and regulates PAI-1 abundance by stabilizing or destabilizing PAI-1 mRNA [111]. This dual function of SERBP1 depends on intracellular localization and cellular context [112]. PAI-1, which is up-regulated in COVID-19 patients diagnosed with CRS, promotes endothelial dysfunction and a hypercoagulable state that predisposes to thrombus formation [113,114]. Taking into account the therapeutic effect of Jusvinza for AR and COVID-19 treatments [22,48], validating whether NCL and SERBP1 down-regulation in Jusvinza-treated neutrophils is related to impairment of mtDNA internalization and decreased PAI-1 levels, respectively, could be of interest. Such molecular events could support the anti-inflammatory and anticoagulant effects of Jusvinza [24].

Remarkably, an array of proteins related to nucleosome organization were identified at 6 h and 18 h after Jusvinza treatment. This cluster of proteins included members of the histone family (HIST1H1B, H1Fx, and HIST1H1E) which were down-regulated in the presence of Jusvinza. Histone H1 regulates nucleosome condensation into chromatin fibers. Citrullination of histone H1 mediated by the enzyme peptidylarginine deiminase 4 (PAD4) results in histone H1 displacement from chromatin and global chromatin decondensation; this event is required for extracellular chromatin release during NET formation [115]. As part of the innate immune response, NETs play a beneficial role in pathogen trapping during infection. However, excessive production and/or inappropriate NET removal drive the severe inflammation that characterizes NET-related pathologies like autoimmune diseases and severe COVID-19 [116]. Histones and high-mobility group protein B1 (HMGB1), which was also down-regulated in Jusvinza-treated neutrophils, are NET structural components [116]. HMGB1 is a nuclear chromatin-associated non-histone protein which is also localized in the extracellular environment [117]. Extracellular HMGB1 functions as a danger-associated molecular pattern (DAMP), amplifying the immune response. Through binding to multiple surface receptors (TLR2, TLR4, RAGE, and CXCR4), alarmin HMGB1 leads to downstream NF-κB activation, neutrophil chemotaxis, and NET formation [117,118]. In fact, HMGB1 has been proposed as a potential therapeutic target for severe inflammatory diseases [119,120]. The concomitant down-regulation of NET structural components in the presence of Jusvinza indicates that the peptide could circumvent the inflammatory process mediated by the formation of NETs. Importantly, such a finding was corroborated by in vitro experiments, in which Jusvinza was shown to decrease NET formation in cultures of neutrophils isolated from healthy donors and stimulated with LPS. One limitation of this study is that NETosis was detected by only one method, even though it has been widely reported by several groups [121,122,123]. However, this method was useful in our research, since we could observe how the addition of LPS to neutrophil cultures led them to fall into NETosis. Furthermore, we verified that Jusvinza does not induce NETosis per se and that the addition of Jusvinza with LPS—and even 30 min after adding LPS—avoids the development of NETosis. These results confirm that Jusvinza can be useful for treating diseases associated with an increase in NETosis beyond RA.

## 5. Conclusions

**Figure 6:**
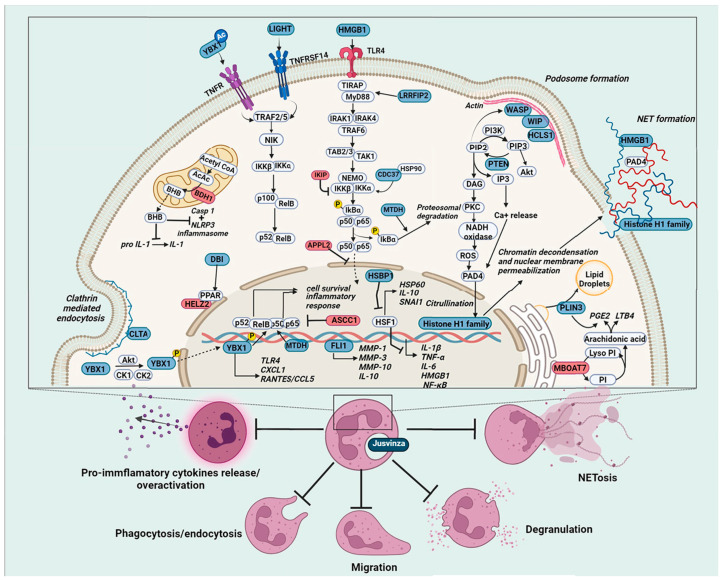
Molecular basis of Jusvinza’s mechanism of action on neutrophils. Jusvinza treatment inhibits pro-inflammatory cytokine release and NETosis. Supporting Jusvinza’s anti-inflammatory effect, the proteomic profile includes several transcription factors (YBX1 and FLI1) and regulator proteins of the heat shock response (HSBP1) and the NF κB signaling pathway. The abundance levels of positive regulators of NF-κB signaling (LIGHT, MTDH, HMGB1, CDC37, and LRRFIP2) were decreased, while negative regulators (APPL2, IKBIP, and ASCC1) were increased in Jusvinza-treated neutrophils. Structural components of NETs (histones and HMGB1) were also decreased in response to Jusvinza treatment. Furthermore, the peptide could inhibit PAD4 activation dependent on Ca^2+^/ROS and consequently suppress NET release. Additionally, proteins related to lipid metabolic pathways (DBI, HELZ2, MBOAT7, and PLIN3) were identified, some of which regulate the synthesis of lipid metabolites (LTB4 and PGE2) with immunoregulatory properties. Juzvinza treatment also modulates proteins related to neutrophil effector functions, such as phagocytosis/endocytosis (CLTA), migration, and priming of neutrophils (HCLS1, WIP, and WASP), probably decreasing the over-activation of such cells in chronic inflammatory conditions. Proteins are represented as boxes and colored according to their expression levels (blue, decreased; red, increased; grey, not identified). In signaling pathways, the lines indicate regulatory events (arrows: activation, lines: inhibition).

Our proteomic analysis and the results of clinical trials in RA [8] and clinical studies in COVID-19 patients [22,23,124] treated with Jusvinza provided data indicating that this drug modulates the effector functions of neutrophils. The peptide could negatively regulate the transcription factor NF-kB by reducing the expression of positive regulators in this signaling pathway while increasing the levels of negative regulators (Figure 6). Furthermore, Jusvinza could inhibit the activation of PAD4 enzymes, with consequent inhibition of NETs. Clinical studies have shown that Jusvinza reduces levels of anti-CCPs (cyclic citrullinated peptides) in RA patients after six months of treatment. Anti-CCPs are strongly implicated in the pathogenesis of RA [8]. The main source of PAD enzymes that citrullinate self-proteins is NETosis [124].

On the other hand, proteins associated with neutrophil migration and phagocytosis showed decreased expression in the proteomic profile (Figure 6). This finding supports the idea that Jusvinza could reduce the over-activation of neutrophils. In severe COVID-19 patients treated with Jusvinza, reduced levels of ferritin, D-dimer, and fibrinogen were observed. These three biomarkers are associated with thrombus formation, with excessive NETs (neutrophil extracellular traps) playing a critical role in coagulopathy and immunothrombosis [125,126]. In addition, Jusvinza treatment modulates several proteins associated with lipid metabolic pathways that have anti-inflammatory properties. This observation is consistent with the protective effects of Jusvinza against oxidation and degradation of APOA1 [7,68].

In summary, the proteomic analysis presented here provides evidence supporting the anti-inflammatory and immunoregulatory properties of Jusvinza. Additionally, it suggests that Jusvinza could be beneficial for treating diseases characterized by hyperinflammation, including autoimmune conditions (such as lupus, psoriasis, and arthritic psoriasis), atherosclerosis, and type II diabetes.

## Figures and Tables

**Table 1:** Demographic characteristics of rheumatoid arthritis patients.

RA Patients	Sex	Age	Ethnicity	DAS 28 *
1	Female	50	White	4.98
2	Female	58	White	4.82
3	Female	59	White	4.99
4	Female	50	White	4.85

* DAS: Disease Activity Score.

## Data Availability

Data are contained within the article or the Supplementary Materials.

## Supplementary Materials

The following supporting information can be downloaded at: https://www.mdpi.com/article/10.3390/biomedicines12122740/s1, Figure S1: Protein clusters according to their expression patterns. (A) The 149 proteins differentially modulated by Jusvinza at 6 h or 18 h were grouped into six clusters using the k-means algorithm implemented in MeV (MultiExperiment Viewer). (B) Heatmap visualization of the protein clusters using the Matrix2png software tool. The color representing the proteins is proportional to the fold change in Jusvinza-treated neutrophils at 6 h or 18 h. The grey color represents unidentified proteins; Figure S2: Cellular components significantly represented in the proteomic profiles (*p*-value < 0.01, enrichment factor > 1.5) modulated in neutrophils treated with Jusvinza after 6 h and 18 h. The analysis was performed using the Metascape gene annotation and analysis resource (https://metascape.org/, accessed on 5 November 2020). In the heatmap, enriched terms are colored according to *p*-values; Table S1: Proteomic profile of neutrophils treated with Jusvinza. Proteins differentially modulated by Jusvinza in neutrophils isolated from four and at least three RA patients; Table S2: Biological processes, pathways, and cellular components significantly represented (*p*-value < 0.01, enrichment factor > 1.5) in the proteomic profile modulated by Jusvinza at 6 h and 18 h in neutrophils isolated from four RA patients. The functional enrichment was performed using the Metascape gene annotation and analysis resource (https://metascape.org/, accessed on 5 November 2020).
