# Supplementary figures and images for: Proteomic Profile Regulated by the Immunomodulatory Jusvinza Drug in Neutrophils Isolated from Rheumatoid Arthritis Patients
Source: Biomedicines. 2024 Nov 29;12(12):2740. doi: 10.3390/biomedicines12122740 (PMC11727316; doi:10.3390/biomedicines12122740)

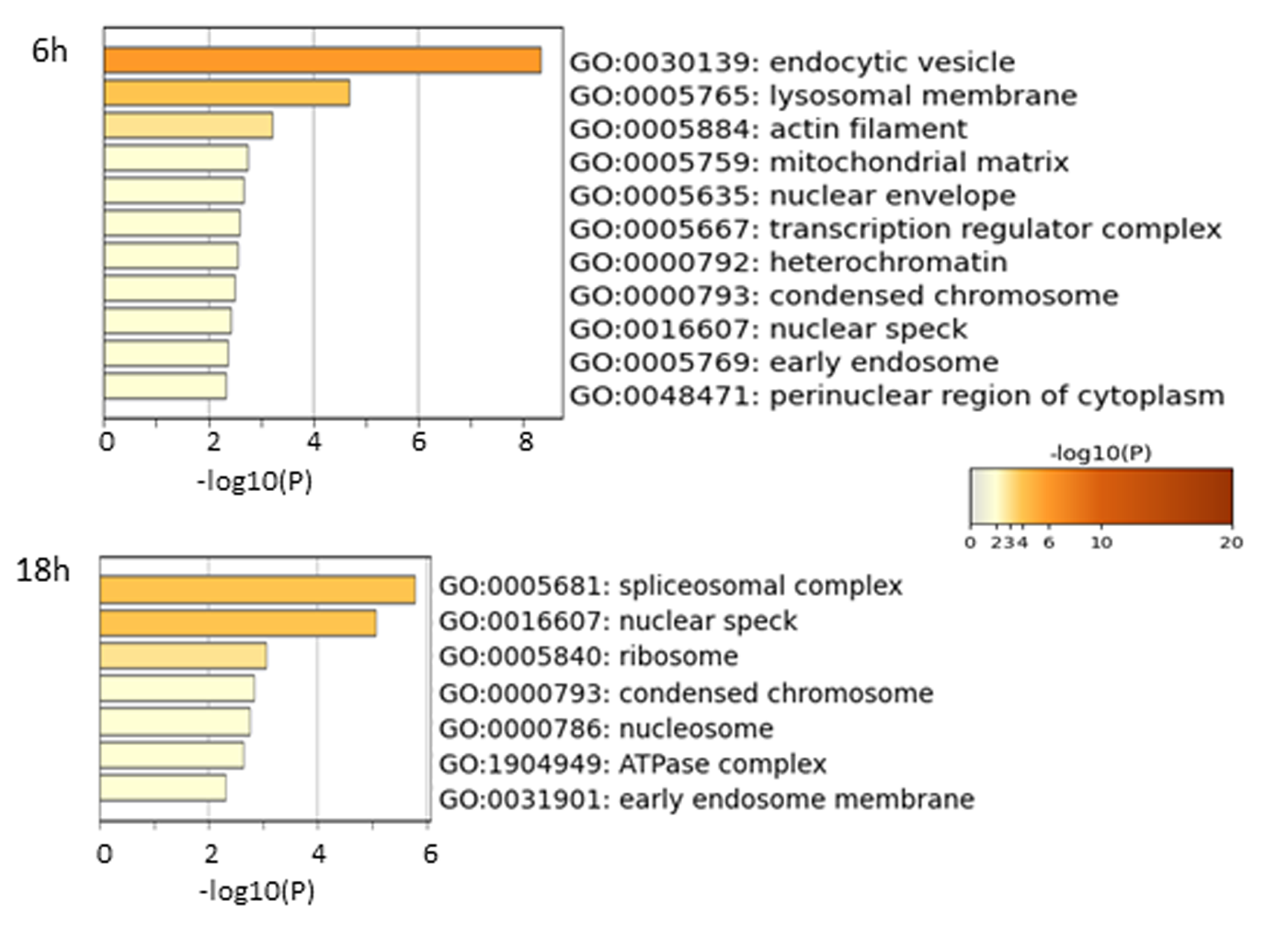

Supplement: Supplementary file 1 [file biomedicines-12-02740-s001.zip › Figure_S2.tif]

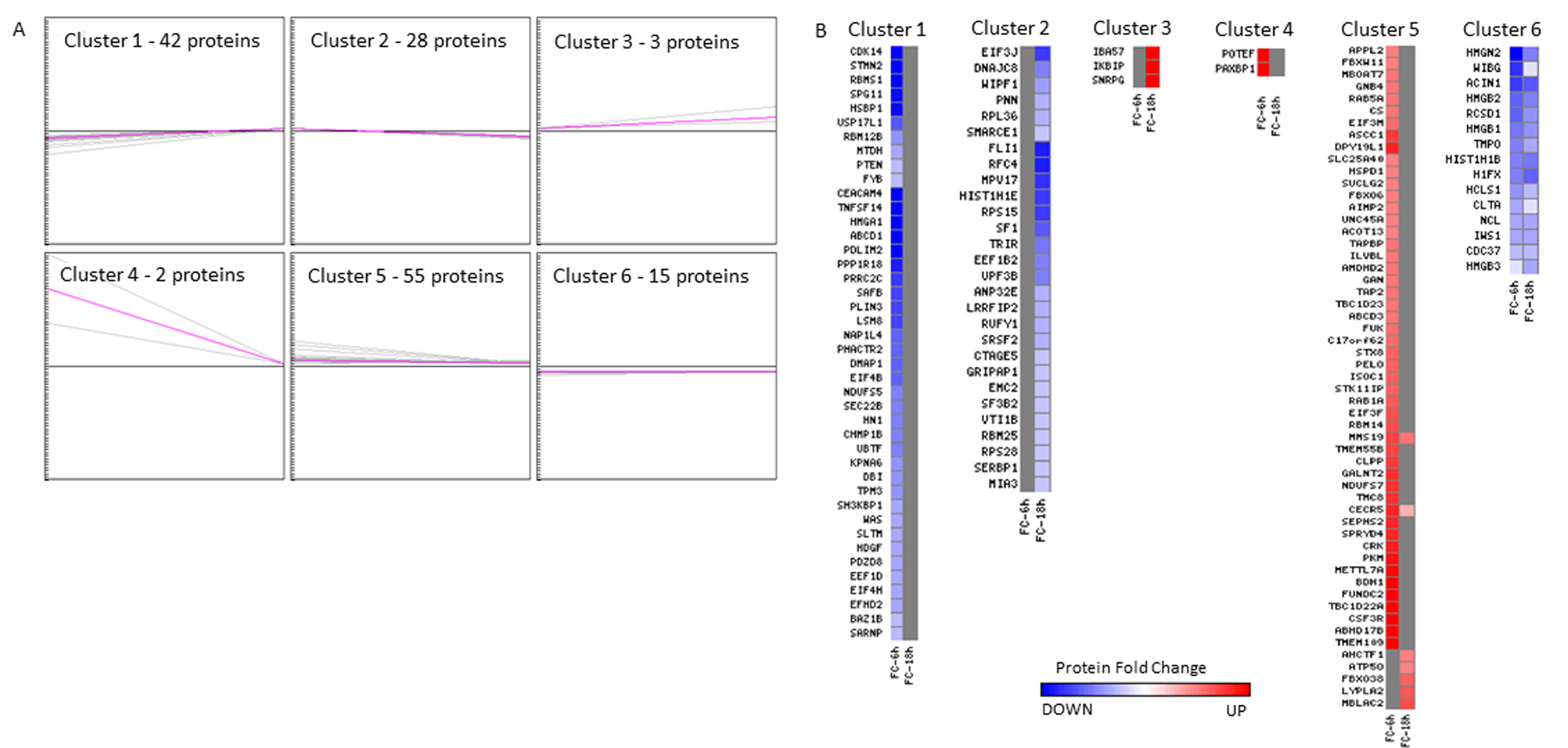

Supplement: Supplementary file 1 [file biomedicines-12-02740-s001.zip › Figure_S1.tif]
